# Natural history of gastric cancer—a case followed up for eight years: early to advanced gastric cancer

**DOI:** 10.1007/s12328-012-0325-2

**Published:** 2012-07-29

**Authors:** Junko Fujisaki, Toshifusa Nakajima, Toshiaki Hirasawa, Yorimasa Yamamoto, Akiyoshi Ishiyama, Tomohiro Tsuchida, Etsuo Hoshino, Masahiro Igarashi, Toshiharu Yamaguchi

**Affiliations:** 1 Endoscopy Division, Gastrointestinal Center, Cancer Institute Hospital, 3-10-6 Ariake, Koto-ku, Tokyo, 135-8550 Japan; 2 Department of Gastroenterological Surgery, Cancer Institute Hospital, 3-10-6 Ariake, Koto-ku, Tokyo, 135-8550 Japan

**Keywords:** Natural history, Gastric cancer, Endoscopic findings

## Abstract

We experienced a case of gastric cancer that was prospectively followed up for 8 years. With severe heart disease, the patient did not wish surgery or anticancer drug treatment. After informed consent was obtained, he was followed up for 8 years. He received upper gastrointestinal endoscopy every year, which revealed IIc early gastric cancer, and biopsy showed well differentiated adenocarcinoma. A flat and mildly depressed lesion with redness was observed on endoscopy, exhibiting typical morphology of IIc-type early gastric cancer. The appearance of IIc M cancer was observed macroscopically from 2000 to 2003. Four years later, surface irregularity with ulceration appeared. Then, the whole lesion was elevated, which suggested submucosal invasion, and the tumor exhibited the morphology of IIa + IIc or type 3. The ulcer became deeper and elevated boundaries were formed. Horizontal expansion of the flat lesion was mild, while invasion to deeper layers was predominant. Eventually, he died of heart failure. Estimated M cancer was observed for about 3 years, followed by invasion to deeper layers. Taken together, this is a valuable case that followed up the manner of invasion to deeper layers over time from early to advanced gastric cancer.

## Introduction

Several reports have shown the process from early to advanced gastric cancer, and macroscopic morphological progression has been observed by retrospective image analysis. However, a prospective follow-up of gradual morphological changes is markedly rare because the conditions allowing such a follow-up are limited [1]. In general, gastric cancer remains in the mucosa for a long time and once it infiltrates to the submucosal layer the growth speed is accelerated and the tumor develops to advanced cancer. We here report the progression process in a case followed up over time from early to advanced gastric cancer and discuss the growth and development pattern.

## Case report

### Present history

An 89-year-old male complaining of epigastric pain underwent upper gastrointestinal endoscopy. A shallow depressed lesion with redness was observed at the anterior wall of the upper gastric body, and IIc-type early gastric cancer was diagnosed. Biopsy revealed differentiated adenocarcinoma. There was no abnormality in laboratory data. Due to severe heart complications (pacemaker implanted), surgical resection of gastric cancer was not feasible. The ASA physical status classification system gave category 3. Due to his old age and complications, he and his family hoped for a periodic follow-up without intensive treatment. Endoscopic treatment was recommended, but was rejected by him and his family. Endoscopy was carried out every 6 months to 1 year as a follow-up.

### Past history

Pacemaker implant for arrhythmia at 85 years old.

### Laboratory data

No abnormality other than mild anemia.

### Clinical course

In 1999, at 89 years old, he complained of mild epigastric pain at the clinic visit, but there was no physical finding. Abdominal ultrasonography and abdominal computed tomography revealed liver cysts but no swollen lymph nodes. Due to severe heart disease, it was decided that he was unable to undergo surgery, and he had been followed up periodically since then. Laboratory data showed no abnormality.

In 1999, CA19-9 and CEA showed normal values of 3.2 and 9.19, respectively, but CA19-9 was slightly elevated since 2005 and it was 376.3 in 2007. He was confirmed dead due to heart failure in December 2007 at 97 years old.

### Endoscopic time-course change

**Fig. 1:**
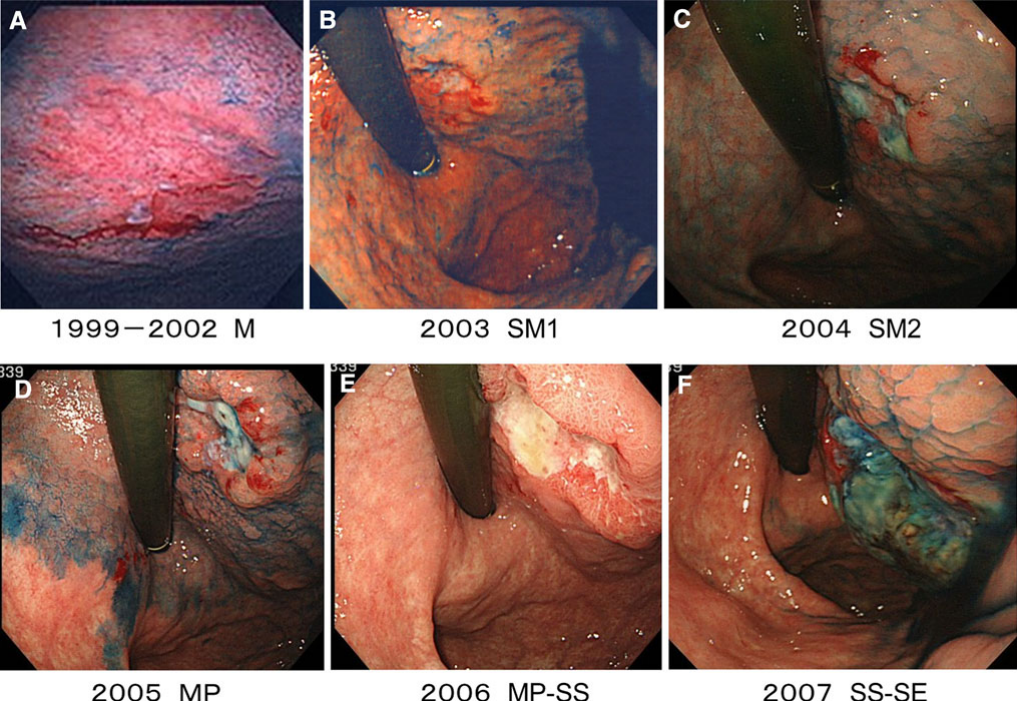
Endoscopic findings of gastric cancer natural course. **a** Slightly depressed lesion with redness was found at the upper body of the stomach. These findings were unchanged for 2 years. **b** Elevated boundary around the lesion was detected. **c** Elevated boundary around the lesion was marked and depressed site became deeper. **d** Size became larger with deep ulcer formation in the center of the lesion. **e** The lesion slightly enlarged to 25 mm. In 2006, the size of the lesion and the ulcer area enlarged. **f** The size increased to 40 mm. The ulcer area and elevated boundaries enlarged even more. **g** Ulcer and elevation around the lesion became marked

Between 1999 and 2002, a shallow depressed lesion 2 cm in size was confirmed at the anterior wall of the upper gastric body. Invasion depth M was suspected. The morphology of the IIc lesion found in 1999 had remained constant until 2002. The lesion was a little smaller than 20 mm in size (Fig. 1a).

Furthermore, 3 years later, endoscopy showed irregularity on the depressed surface with elevated boundaries, which suggested submucosal invasion (Fig. 1b).

In 2004, the ulcer area enlarged and elevated boundaries appeared. Invasion depth SM2 was suspected (Fig. 1c) [2]. Despite the elevated boundaries, the size of the tumor remained constant and a little larger than 20 mm.

In 2005, the ulcer became deeper and the boundaries were even higher. Invasion depth MP was suspected (Fig. 1d). The lesion slightly enlarged to 25 mm. In 2006, the size of the lesion and the ulcer area enlarged. Invasion depth MP–SS was suspected (Fig. 1e). The size increased to 40 mm. In 2007, the ulcer area and elevated boundaries enlarged even more. Invasion depth SS–SE was suspected (Fig. 1f). The size was about 60 mm.

**Fig. 2:**
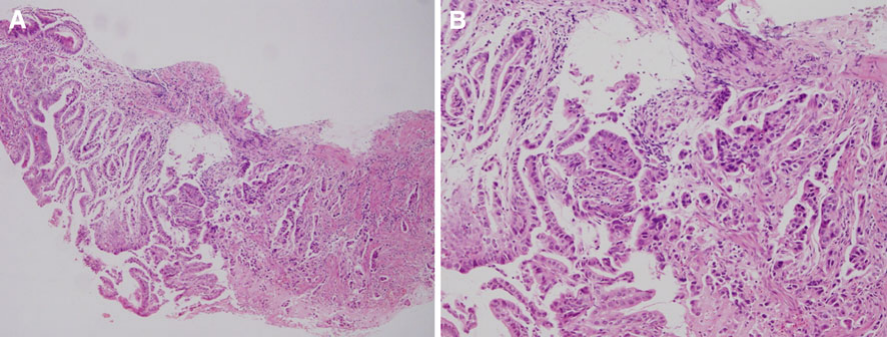
**a** Biopsy specimen revealed well to moderately differentiated adenocarcinoma. **b** High-power view of biopsy specimen

A biopsy specimen showed well to moderately differentiated adenocarcinoma in 2002 (Fig. 2a, b).

Images exhibiting early cancer were observed between 1999 and 2002 and thereafter the ulcer was deeper with heightened boundaries, which suggested the tendency of deeper invasion. Morphological change after submucosal invasion was striking. Eventually, no metastasis was found by imaging examinations but an increase in CA19-9 suggested lymph node metastasis. However, gastric cancer did not become the direct cause of death.

## Discussion

There have been a number of retrospective studies on morphological changes from early to advanced gastric cancer. When the lesion becomes larger, the proportion of the lesion with deeper invasion increases. Considering this, the growth speed of small early gastric cancer is slow and once it exceeds 4 cm in size and infiltrates to the proper muscle layer, it typically enlarges rapidly in a short period of time. Nakamura et al. [3] reported that it took 7 years on average to progress from M to SM cancer and 2–3 years from SM to advanced cancer. In the present case, it is unclear when an intramucosal lesion first appeared, but the lesion remained within the mucosal layer at least for 4 years from the first detection, and it was suggested that endoscopic mucosal resection was indicated at that time.

The tumor invaded to SM and to MP, and it took 3 years to exhibit the appearance of advanced cancer, which was consistent with previous reports. Tsukuma [4] reported that early gastric cancer showed a relatively long natural history in general and the median duration of those who remained in the early stage was estimated as 44 months. Since 2005 when the tumor was confirmed to be advanced cancer, it had dramatically enlarged to a size twice that 1 year previously by endoscopy.

As factors influencing the growth of early gastric cancer, age, morphological types, presence of ulcer, and histology have been reported. Retrospective studies on gastric cancer revealed that IIc-type gastric cancer was the most frequently observed early lesion, the period that IIc lesions remained M cancer was long and ulceration was the cause of slow growth.

It was reported that growth was accelerated as tumors developed close to advanced cancer [4]. Since most colorectal cancers exhibit an elevated type and it is easier to analyze them by X-ray retrospectively, it is possible to calculate the growth speed. Matsui et al. [5] reported the results of an X-ray study that showed small colorectal cancer developed according to certain exponential functions (diameter = 12.5 × 2^*t*/77^ or volume = 1 × 10^3 ^× 2^*t*/26^), where *t* is the time in months. According to this equation, the doubling time of early colorectal cancer was calculated as 26 months. Mucosal cancer invaded to SM and the growth was then accelerated in colorectal cancer [6]. In general, tumor doubling time = (days × log2)/[3 × log (tumor diameter at this time/tumor diameter at former time)]. According to this calculation, the doubling time of this tumor was 298 days.

On the other hand, it is known that fibrosis arising from repeated ulceration and scarring in a malignant cycle inhibits the growth of gastric cancer, and SM cancer grows faster than M cancer. Judging from the size, the tumor remained 20–25 mm in size for 3–4 years, and then enlarged threefold in 3 years.

In the present case, although the tumor had been early gastric cancer 8 years before, it grew to advanced cancer in 8 years. Endoscopic findings suggest that the tumor was M cancer in 1999 when it was first detected. In 2003, it showed a change and resembled a submucosal tumor (SMT), which indicated SM infiltration [2]. Then, the SMT-like elevation was recognized as elevated boundaries and it was diagnosed as advanced cancer in 2005. Endoscopic findings showed that the tumor showed the morphology of early gastric cancer for about 5 years, and then developed to advanced cancer. Lymph node metastasis seemed to be from 2005, as CA19-9 was gradually increased from that time. Hirose et al. [7] reported that peripheral venous CA19-9 levels were significantly higher in patients with positive lymph node staining with immunohistochemical study than in those without it. Since early gastric cancer remained at the early stage for about 8 years, M cancer detected 7 years previously could have been treated by endoscopic resection.

Recently endoscopic treatment has been widely employed for early gastric cancer, in particular small gastric cancer, while the necessity of the treatment is discussed. In Japan, an aging society, the detection rate of early gastric cancer in the elderly is high. In the elderly, differentiated adenocarcinoma is often observed because it is induced by infection with *Helicobacter pylori*. Although the necessity of treatment is often discussed, conclusions have not been reached, because estimation of life expectancy is difficult. Considering the natural history of gastric cancer, the period of gastric cancer which remains in the mucosa is long. Considering the growth curve of the intramucosal lesion, it is necessary to deal appropriately with each case, such as a cut end-positive case, and it is suggested that follow-up is a reasonable option.

Endoscopic treatment should have been possible until 1999, but it was impossible in 2003. However, no appetite loss due to bleeding from the lesion or stenosis was observed and the macroscopic appearance revealed early gastric cancer for as long as about 5 years. Even after advanced cancer was diagnosed, vertical development, rather than horizontal expansion, was mainly observed.

Periodic annual follow-ups allowed observations of macroscopic time-course changes in invasion of the gastric cancer to deeper layers. Time-course changes in invasion to deeper layers were expressed as the height of elevated boundaries. Although there are a number of reports on the natural history of gastric cancer, they are mostly retrospective reports and the change is estimated based on the endoscopic pictures at the first detection and several years previously. This is a rare and valuable case report that periodically followed up growth and morphology of gastric cancer for 8 years and showed the growth pattern of gastric cancer in detail.
